# Unraveling an Unusual Presentation of Parotid Malignancy: A Case Report

**DOI:** 10.7759/cureus.61602

**Published:** 2024-06-03

**Authors:** Shivangi Tiwari, Gayatri Muley, Yashika Gupta, Najmeh Mirkhushal, Pratik Maske

**Affiliations:** 1 General Surgery, Grant Government Medical College, Mumbai, IND

**Keywords:** malignant, benign, unusual, parotid gland, mucoepidermoid carcinoma

## Abstract

Mucoepidermoid carcinoma (MEC) is a salivary gland tumor commonly arising from the parotid gland. MEC has various presenting symptoms, including a painless, slow-growing mass below or anterior to the ear lobule. However, an unusual presentation can also be in the form of post-auricular swelling. Other more common benign differentials for post-auricular swelling include lymphadenopathy, epidermoid cysts, and lipomas. Thus, diagnosing a postauricular swelling as MEC solely based on clinical presentation is challenging, and a high suspicion, as well as a multidisciplinary approach with various radiological investigations such as computed tomography (CT) and magnetic resonance imaging (MRI), are required in collaboration with histopathological assessment for an accurate diagnosis of this malignancy. Prognosis depends on various factors, including the grade of the tumor, the patient's age, and comorbidities, as well as the stage at the time of diagnosis. Early diagnosis and surgical intervention are the mainstays of treatment, which can be followed by adjuvant radiotherapy based on the stage of the malignancy. This is a report of a patient who presented with post-auricular swelling, which was initially misdiagnosed as a benign necrotic lymph node. After further evaluation, it was found to be a mucoepidermoid carcinoma of the parotid gland, which was managed by surgical excision and radiotherapy.

## Introduction

Mucoepidermoid carcinoma is the most common malignant tumor of the salivary gland and commonly involves the parotid gland [1]. Mucoepidermoid carcinoma (MEC) of the salivary gland is believed to arise from pluripotent reserve cells of the excretory ducts that differentiate into squamous, columnar, and mucous cells [2].

It develops in the salivary glands and is characterized by the presence of three distinct cell types within the tumor: mucinous cells, squamous cells, and intermediate cells. The heterogeneity of the type of cells helps to distinguish MEC from other types of salivary gland tumors as well as leads to an altered course and clinical presentation of the tumor [1].

The typical presentation of MEC involves the development of a painless swelling or mass within the affected salivary gland (65.2%) [3]. Patients may notice a gradual increase in the size of the mass over time. Other symptoms, such as facial weakness or difficulty swallowing, may occur depending on the location and size of the tumor. Management of MEC involves a multidisciplinary approach, with surgery serving as the primary treatment modality.

## Case presentation

**Figure 1:**
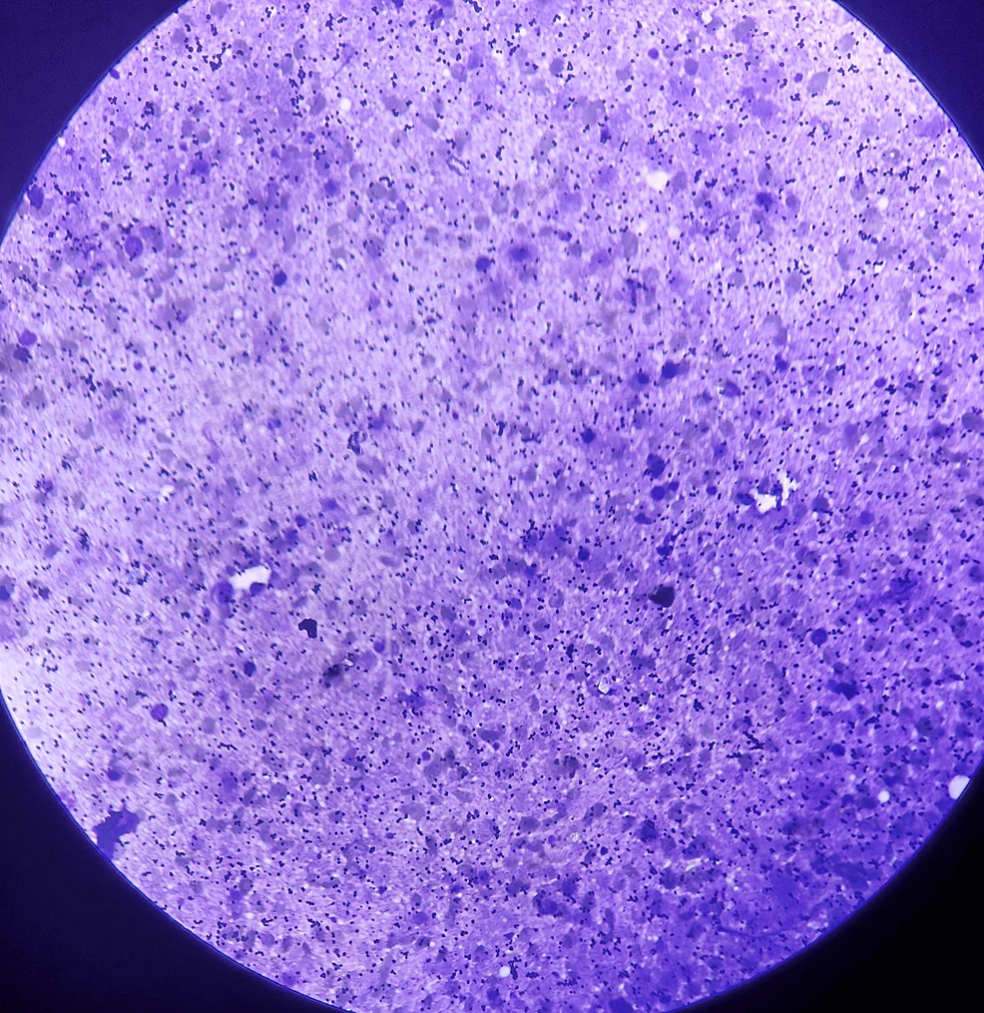
Microscopic image of necrotic tissue seen on fine needle aspiration cytology

A 38-year-old female presented to the General Surgery Outpatient Department with complaints of swelling in the left post-auricular region for the past month, which was gradually progressive in size. On examination, the swelling was 1.5 cm* by 1 cm in size, smooth, firm, and non-tender, with no redness or other skin changes, not fixed to the skin, and no signs of facial nerve involvement. Ultrasonography was suggestive of conglomerated partial necrotic lymph nodes in the post-auricular region. Ultrasonography-guided aspiration of the lesion was done, which was negative for *Mycobacterium tuberculosis*, and histopathological examination revealed necrotic tissue (Figure 1).

**Figure 2:**
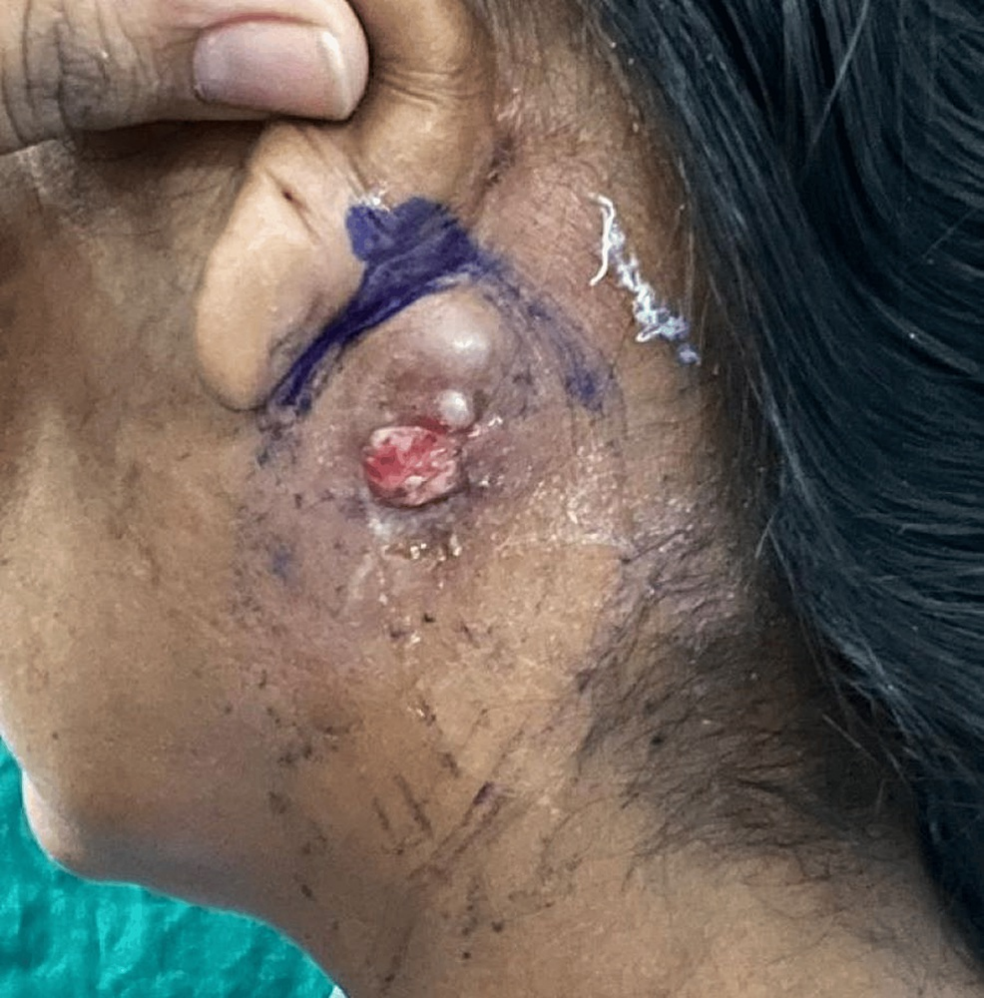
Clinical presentation with recurrent nodules

**Figure 3:**
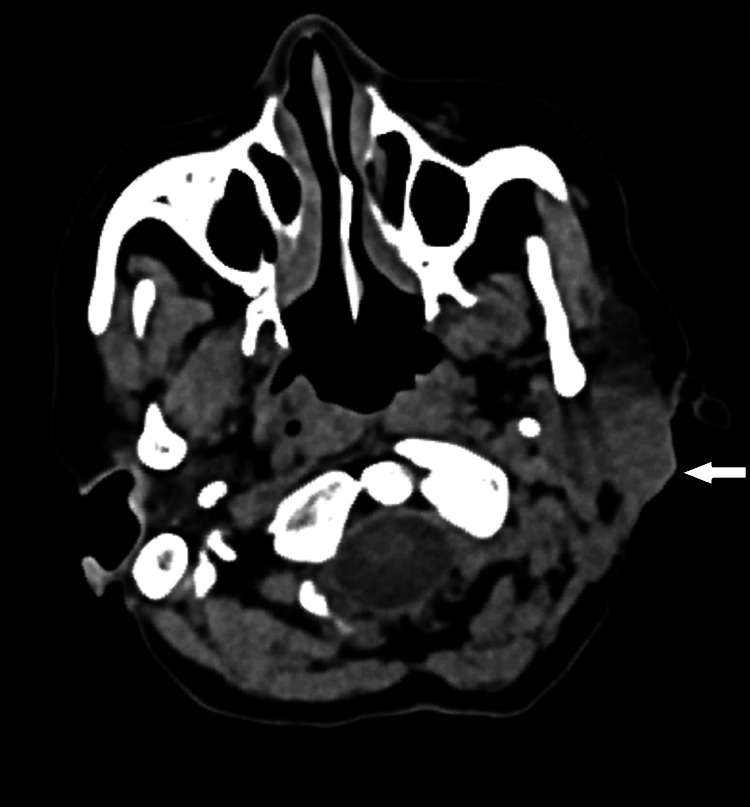
CT image showing the deep and superficial lobes of the left parotid gland with the bulge in the superficial lobe (arrow)

**Figure 4:**
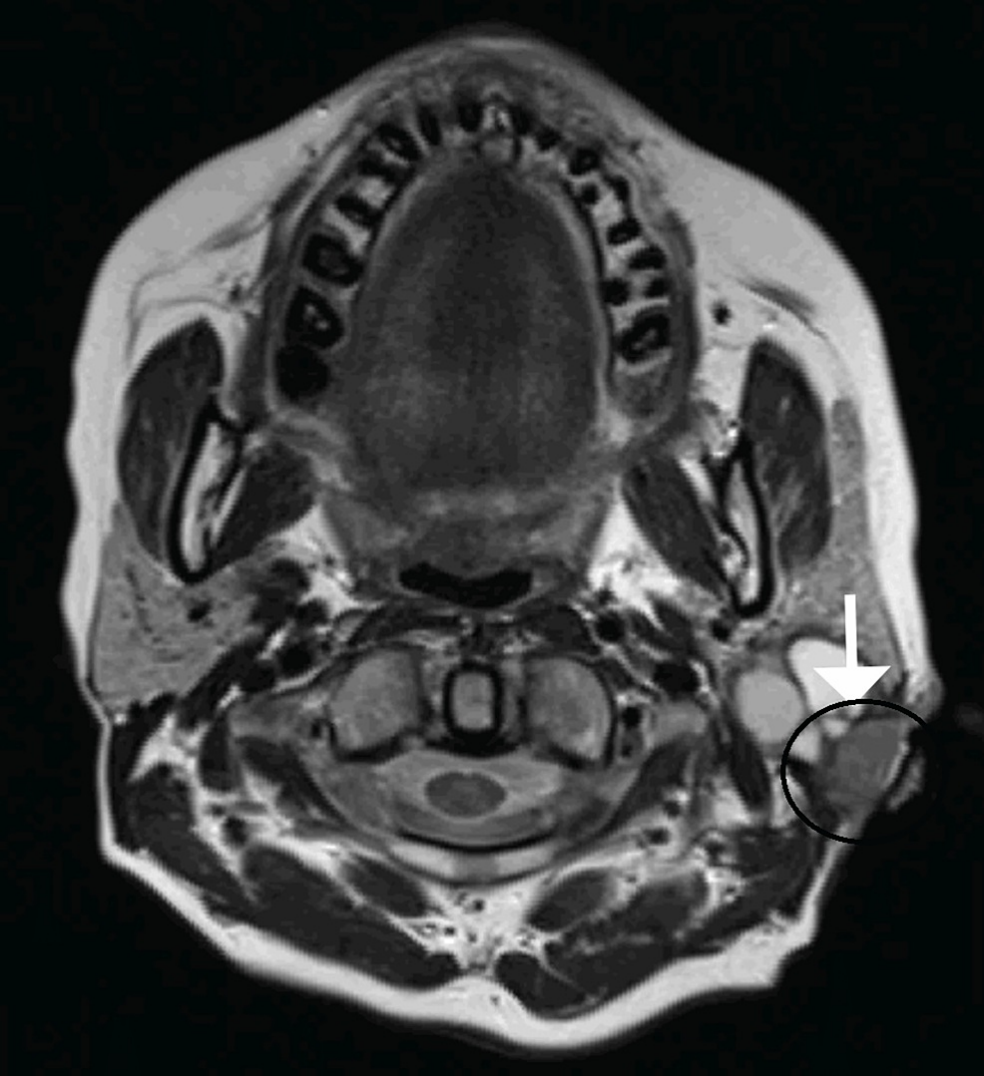
MRI image showing a tumor in the left parotid gland involving the fasciovenous plane of Patey

The decision was taken to go ahead with an excisional biopsy of the lesion. The lesion was excised under local anesthesia and sent for histopathological examination, which revealed a low-grade mucoepidermoid carcinoma. On further follow-up, the patient presented with a recurrent nodule and an ulcer in the post-auricular region three weeks following the excisional biopsy (Figure 2). Computed tomography of the neck revealed a multi-lobulated mixed-solid cystic lesion with its epicenter in the fasciovenous plane of Patey without involvement of the deep lobe of the parotid gland, with sub-centimetric reactive lymph nodes level Ib and II on the left side (Figure 3). Further, magnetic resonance imaging revealed a similar finding without invasion of the tumor into the facial nerve (Figure 4). The decision was taken to proceed with a superficial parotidectomy. 

**Figure 5:**
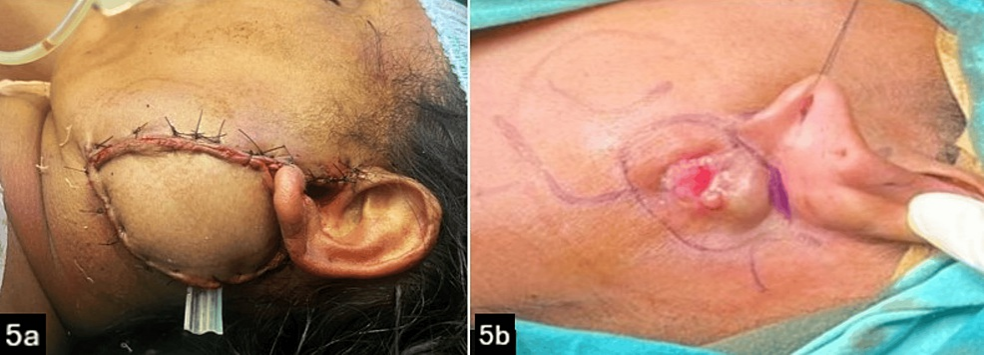
5a: Image showing Pectoralis Major Myocutaneous Flap 5b: Image showing extended lazy-S incision

**Figure 6:**
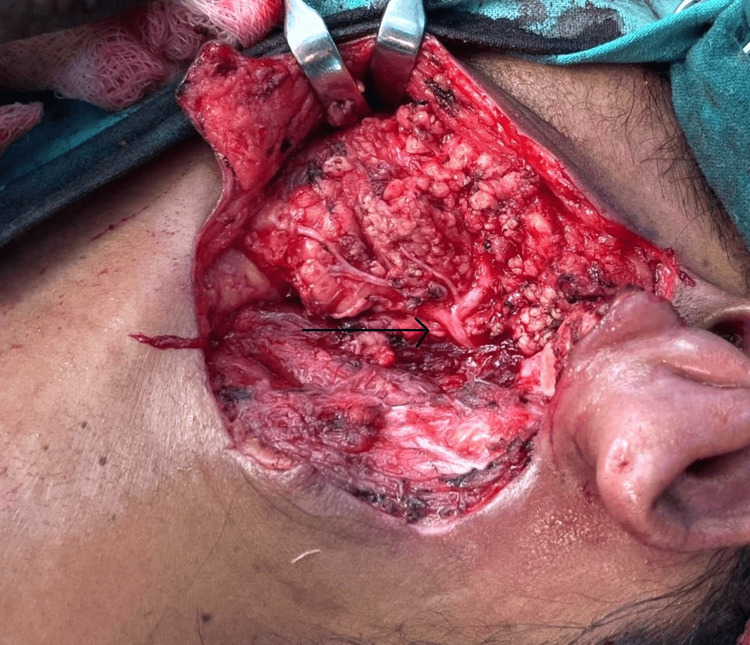
Intraoperative image showing the facial nerve and its branches (arrow)

An extended lazy S incision (Figure 5b) was taken for surgical removal of the superficial lobe of the left parotid gland, including the previous scar, as well as the recurrent nodule present at the previous excision site. Intraoperatively, the facial nerve and its branches were identified and preserved (Figure 6). The resected superficial lobe of the parotid was sent for a frozen section. A pectoralis major myocutaneous rotational flap (PMMC) was done to cover the skin defect in the post-auricular region (Figure 5a).

**Figure 7:**
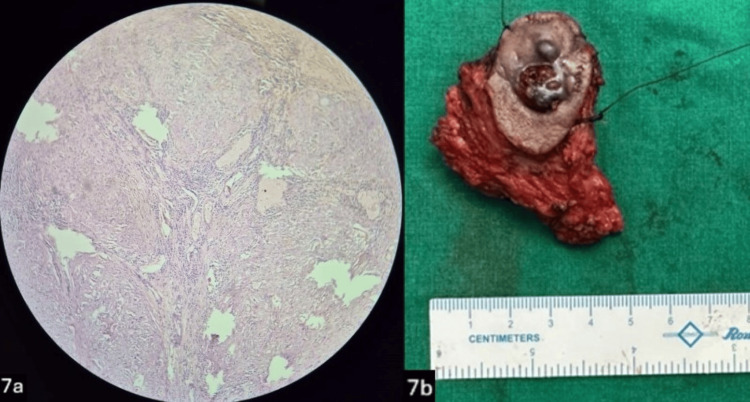
7a Low-powered microscopic image showing epidermal cells with few cystic spaces, suggestive of mucoepidermoid carcinoma. 7b: Resected superficial parotid gland specimen

The flap healed without any postoperative complications. Histopathological examination of the specimen revealed a well-circumscribed tumor with a solid and cystic component suggestive of low-grade mucoepidermoid carcinoma with clear superior, inferior, medial, and lateral margins and involvement of the base (Figures 7a, 7b). The patient was referred for further locoregional radiation therapy.

## Discussion

Mucoepidermoid carcinoma comprises 10-15% of all salivary gland malignancies. Among the salivary glands, the parotid gland, which is the largest of the major salivary glands located in front of the ear, is the most frequent site where mucoepidermoid carcinoma occurs (56.8%) [3]. On average, females are more affected than males, with the mean age of diagnosis being 48-58 years [4,5]. Major prognostic factors include increasing age, comorbidities, high tumor grade, advanced pathologic group stage, and positive surgical margins.

One of the intriguing aspects of MEC is its varied clinical presentation and histological features. Clinically, patients may present with a painless, slow-growing mass in the parotid region [6], which can often be confused with benign lesions. In our case, the patient presented with post-auricular swelling, which was initially misdiagnosed as a benign lymph node. An isolated post-auricular swelling is a rare presentation of MEC [7]. A similar case study discussed a benign-looking postauricular lesion that was misdiagnosed as an epidermal inclusion cyst, which, on further histological examination, was suggestive of MEC [7].

The diagnosis of mucoepidermoid carcinoma (MEC) typically entails utilizing a combination of imaging modalities, such as ultrasound, MRI, or CT scans, in conjunction with a fine needle aspiration biopsy (FNAB) for cytological examination. On USG, MEC lesions of the salivary glands are mostly associated with heterogeneous echotexture, indistinct margins, irregular shape, and the absence of distal acoustic enhancement with cystic areas and calcifications, which may be difficult to differentiate from a superficial necrotic lymph node due to the site of the lesion [8]. Due to its heterogeneous nature, a definitive diagnosis often requires histopathological examination of surgical specimens obtained through excisional biopsy or partial/total parotidectomy.

Histopathologically, it is classified into three grades: low, intermediate, and high, with low grade (48%) being more common than high grade (38.7%) and intermediate grade (13.3%) being the least common [9]. These three histopathological grades are based on the degree of cytological atypia, the amount of cyst formation, and the relative numbers of mucous, epidermoid, and intermediate cells [10]. The extent of surgical intervention depends on factors such as tumor size, grade, and location, as well as the presence of metastasis to regional lymph nodes. In cases of low-grade MEC confined to the parotid gland, a superficial or total parotidectomy may be curative. However, high-grade tumors or those with nodal involvement may necessitate more aggressive surgical resection, including neck lymph node dissection, potentially followed by adjuvant radiotherapy or chemotherapy.

In our case, since the growth was in the faciocutaneous plane of Patey, it was decided to go ahead with a superficial parotidectomy with an on-table frozen section that showed tumor-free margins on all sides except the base. The patient was advised radiotherapy following the healing of the flap for locoregional clearance.

The prognosis for MEC varies widely depending on several factors, including tumor grade, stage, and the presence of adverse histological features such as perineural invasion or lymphovascular invasion. Generally, low-grade MECs have a favorable prognosis with high rates of long-term survival, while high-grade tumors are associated with a higher risk of recurrence and metastasis.

## Conclusions

Among all the salivary gland neoplasms, MEC has been reported to show the highest false negativity with FNAB, likely due to the presence of cystic architecture and varying proportions of epidermoid cells, intermediate cells, and mucocytes. When combined with its varied clinical presentation, extending from a slow-growing painless mass in the parotid region to a fungating mass in the periauricular region with skin metastasis, a high level of suspicion may prevent the misdiagnosis of a potentially high-grade tumor, which should be differentiated from other similar benign lesions.
